# The oesophagus and cough: laryngo-pharyngeal reflux, microaspiration and vagal reflexes

**DOI:** 10.1186/1745-9974-9-12

**Published:** 2013-04-16

**Authors:** Jaclyn A Smith, Lesley A Houghton

**Affiliations:** 1 Respiratory and Allergy Centre, The University of Manchester, University Hospital of South Manchester, Manchester, UK; 2 Division of Gastroenterology and Hepatology, Mayo Clinic, Florida, USA

## Abstract

Gastro-oesophageal reflux disease is generally considered to be one of the commonest causes of chronic cough, however randomised controlled trials of proton pump inhibitors have often failed to support this notion. This article reviews the most recent studies investigating the mechanisms thought to link reflux and cough, namely laryngo-pharyngeal reflux, micro-aspiration and neuronal cross-organ sensitisation. How recent evidence might shed light on the failure of acid suppressing therapies and suggest new approaches to treating reflux related cough are also discussed.

## Introduction

For over 30 years gastro-oesophageal reflux disease (GORD) has been considered to be a cause of chronic cough, either singly or in association with nasal disease and/or asthma [1]. This was initially based on observational studies and small treatment trials of acid suppressants [2-4]. However, it is the experience of many clinicians that although some patients with chronic cough do claim relief from acid-suppressants, the majority do not. One of the difficulties in understanding why this might be the case has been a shortage of high quality evidence. Indeed a recent Cochrane review of various treatments of GORD (H_2_ receptor antagonists, proton pump inhibitors, motility agents, fundoplication or conservative treatments) for chronic cough found that meta-analysis was not possible for most treatments in adults and none in children due to poor trial design and lack of appropriate data [5]. Nonetheless, a number of appropriately designed randomised controlled trials have been completed assessing acid suppression with proton pump inhibitor (PPI) therapy in adults with chronic cough [6-9]. The majority of these trials reported negative findings and an intention-to-treat analysis of the pooled data found no significant difference from placebo control. The reasons for this lack of efficacy of PPI therapy in chronic cough are unclear but possible explanations include:

i. GORD and chronic cough are both common conditions affecting similar populations but without a causal relationship, thus anecdotal reports of responses to acid suppression are just placebo responses,

ii. a variety of criteria have been used for patient selection in studies of acid suppression, which may not have either targeted or allowed the sub-group of chronic cough patients to be identified that were most likely to respond to acid suppression,

iii. the acidity of the refluxate may not be of major importance in the pathophysiological processes linking reflux and cough.

Hence to better understand the association between GORD and cough requires a re-think of the possible mechanisms connecting these conditions, and how these might relate to sub-groups of patients with chronic cough. The aim of this article is to review recent evidence which might shed light on these mechanisms and thus identify more appropriate management strategies and treatment options for these patients along with possible avenues for drug development.

### Possible mechanisms linking cough and reflux: direct and indirect

Mechanisms whereby gastro-oesophageal reflux events can trigger coughing include i) direct stimulation of the nerve terminals responsible for evoking cough either in the larynx (*laryngo-pharyngeal reflux*) and/or bronchi (*microaspiration*) and ii) indirect stimulation by activation of neural pathways linking the oesophagus to the airway (the *oesophageal-bronchial reflex*).

### Direct mechanisms

#### Laryngopharyngeal reflux

The concept that laryngopharyngeal reflux causes upper airway symptoms has gained increasing attention in recent years, despite the challenges of measuring gastro-oesophageal reflux that extends up into the pharynx and larynx. Reflux into the oesophagus is successfully measured by detecting changes in pH associated with the acidity of the reflux events and more recently by combining this with multi-channel intraluminal impedance monitoring (MII/pH), allowing all reflux, whether liquid or gas, acidic or not, to be detected. Impedance rings allow measurement of the changes in conductance that occur with the movement of liquid (low impedance) and gas (high impedance) up into the oesophagus. In the larynx and pharynx reflux measurement using these technologies is more challenging. In the oesophagus, the baseline impedance level remains relatively stable as the impedance rings remain in contact with the oesophageal mucosa. In contrast, the pharynx is an air filled cavity and hence the baseline level of impedance is unstable, fluctuating depending on whether the impedance rings are in contact with the moist mucosa or in air. Moreover differentiating a retrograde reflux event in the pharynx from a swallow can be difficult and for both these reasons agreement is poor between different observers analysing the traces [10]. Despite these difficulties some investigators have reported pharyngeal reflux findings in patients with chronic cough. One study using MII/pH monitoring in the oesophagus and hypopharynx found on average 2 pharyngeal liquid reflux episodes per 24 hrs in 17 patients [11] whilst another found none in a group of 10 patients with chronic cough [12]. The latter study however, did report that these patients exhibit more weakly acid (pH 4–7) ‘gaseous’ pharyngeal reflux events than controls or GORD patients but other investigators have suggested that gaseous pharyngeal reflux cannot be analysed using impedance due to the air in the pharynx [10].

Other techniques used to assess pharyngeal reflux include the Restech system, which detects changes in pH on exposure to aerosol and liquid reflux in the oropharynx and unlike standard pH electrodes can function in air. Unfortunately two studies comparing the Restech with simultaneous oesophageal MII/pH monitoring have found the majority of pharyngeal pH drops are not associated with oesophageal reflux events [13,14]. For example, in 10 patients with chronic cough, of the 39 oropharyngeal acid events detected by the Restech, 43.6% were swallows, 38% were not associated with any oesophageal MII/pH changes and only 18% were clearly reflux events [13]. Indeed swallow artefacts are likely to be a particular problem in patients with chronic cough who frequently sip fluids to suppress their coughing. Lastly, subjective scoring systems to document laryngeal appearances suggestive of LPR have been used but have largely been discredited as the specificity of findings [15] and inter-observer agreement are poor [16-18].

#### Proximal oesophageal reflux

**Figure 1:**
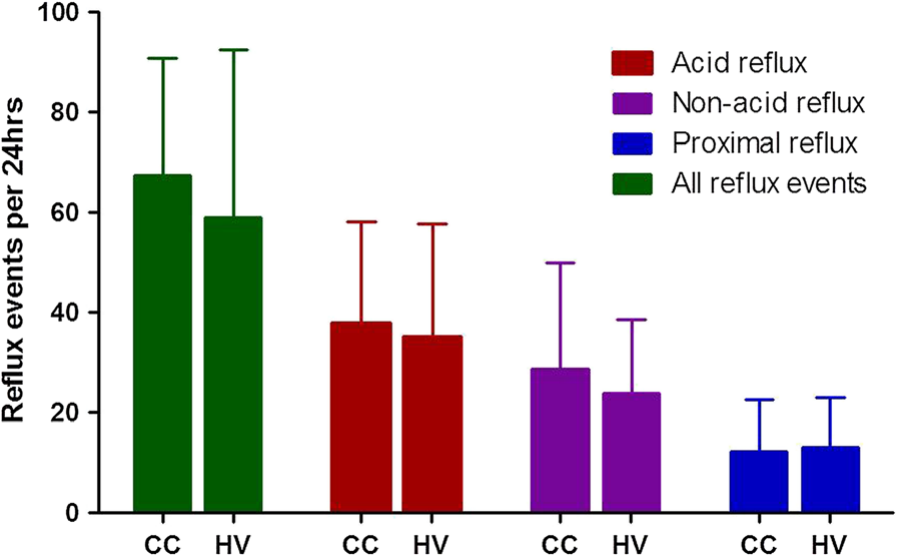
**Numbers of reflux events in a 24 hr period in an unselected group of patients with chronic cough (CC n = 78) and healthy volunteers (HV n = 20). **Total numbers of events are show and the number of acid events (pH < 4), number of non-acid events (pH > 4), and the number of events extending into the proximal oesophagus i.e. 16-18 cm above the lower oesophageal sphincter. Data from [19].

In order to access the larynx and pharynx, refluxate must first traverse the length of the oesophagus and also breach the upper oesophageal sphincter. It could therefore be argued that the presence of excessive reflux in the proximal oesophagus may predispose subjects to LPR. However, the majority of studies both in unselected patients with chronic cough or those in whom extra-oesophageal causes have been excluded have shown that the number and acidity of proximal oesophageal reflux events is no different from healthy controls [19-21], who exhibit a median of 8–9 reflux events per 24 hours (95th percentile 30–31 events) [22,23], see Figure 1. Only one study in a mixed group of patients with typical and atypical reflux symptoms, some of whom also had chronic cough, found greater acid exposure in the proximal and distal oesophagus of chronic cough patients compared with those without cough [24]; but the average oesophageal acid exposure was still within normal limits. Perhaps more importantly we have found no correlation between the number of proximally occurring reflux events [19] and the frequency of coughing, suggesting that the proximal extent of reflux events may not be important for provocation of cough.

#### Upper oesophageal sphincter function

Failure of the upper oesophageal sphincter (UOS) may also increase the risk of refluxate reaching the larynx/pharynx and two recent studies have investigated the role of the UOS in cough. In a healthy control study, voluntary coughing evoked a dramatic contractile response of the UOS, assumed to be a protective reflex response to prevent refluxate entering the pharynx [25]. Interestingly, although this response was preserved in older healthy volunteers (age > 70), the resting UOS pressure was lower, suggesting a possible increased vulnerability to LPR outside cough events. The second study was a retrospective review of patients with chronic cough undergoing high resolution manometry and reflux monitoring, and showed that although resting UOS pressures were within normal limits, 9/34 subjects demonstrated a pathologically increased residual or intra-bolus pressure at the UOS on swallowing [26]. Although it might be speculated that this might increase resistance to pharyngo-oesophageal flow, its clinical significance remains to be established. This study and several others have also reported ineffective oesophageal peristalsis in chronic cough [27,28], with one study finding twice the prevalence of ineffective peristalsis in chronic cough compared with heartburn patients [28]. The presence of ineffective motility in chronic cough was equally as likely in patients with and without acid reflux [27], suggesting that dysmotility was not caused by abnormal oesophageal acid exposure. However, this does not exclude the possibility that poor or delayed oesophageal clearance of refluxate might contribute to the risk of laryngeal and pharyngeal exposure to refluxate or provocation of a broncho-oesophageal reflex.

#### Micro-aspiration

**Figure 2:**
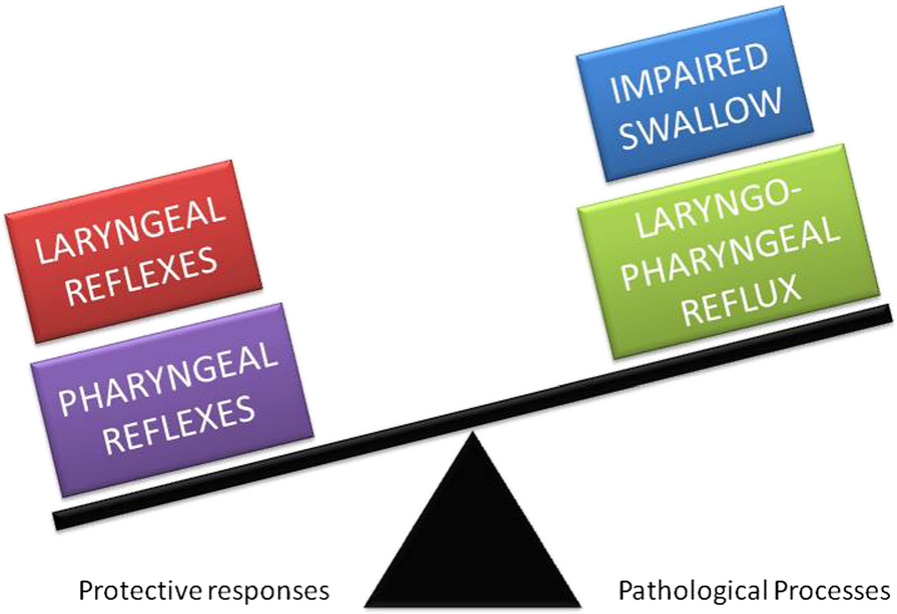
Factors relevant to micro-aspiration of food and refluxate into the airways; laryngeal reflexes include cough and the laryngeal adductor reflex whereas pharyngeal reflexes are reflexive pharyngeal swallow, the pharyngo-glottal closure reflex and the pharyngo upper oesophageal sphincter contractile reflex.

The risk of food or refluxate entering the airway is likely to be a balance between the complex reflex responses that have evolved to protect the airway and pathological processes that increase pharyngeal/laryngeal exposure to food and refluxate, such as impaired co-ordination of swallowing, excessive laryngo-pharyngeal reflux and perhaps also oesophageal dysmotility, see Figure 2. Therefore microaspiration may occur as a consequence of either impairment of the protective reflexes or excessive challenges to these reflexes.

#### Protective pharyngeal and laryngeal reflexes

Studies infusing water into the hypopharynx of healthy controls show that several protective reflexes (reflexive pharyngeal swallow, the pharyngo-glottal closure reflex and the pharyngo-upper oesophageal sphincter contractile reflex) are all provoked well before volumes are reached that are likely to spill into the airway [29]. Although similar studies have yet to be performed in patients with chronic cough, it is interesting to note that healthy volunteers only cough when fluid enters the larynx. In patients with GORD, defined by abnormal oesophageal acid exposure and/or oesophagitis [30], glottal closure and/or swallow responses are evoked by much smaller hypopharyngeal infusion volumes than in healthy controls, whether acid or saline, suggesting a general hypersensitivity to pharyngeal fluid. Interestingly the same GORD subjects exhibited diminished glottal closure in response to laryngeal puffs of air (the laryngeal adductor reflex) compared with controls, implying that whilst these patients have exaggerated pharyngeal reflexes, laryngeal responses may be impaired. The consequent risk of microaspiration in these subjects is difficult to extrapolate. The same investigators also studied the laryngeal adductor reflex in patients with chronic cough associated with GORD, again defined by abnormal oesophageal acid exposure and/or oesophagitis, with some improvement of cough with previous acid suppression therapy [31]. As with the GORD patients without cough, the chronic cough/GORD patients had diminished laryngeal adductor responses compared with healthy controls. Whether the laryngeal adductor reflex is abnormal in chronic cough patients without GORD and whether this deficit has any pathophysiological consequences in the context of excessive coughing remains unknown.

Reflex glottal closure has also been studied in response to inhalation of the chemical irritant ammonia, measuring the threshold for evoking a ‘glottic stop’, which is a rapid transient glottal closure which can be detected by a sudden drop in inspiratory flow during inhalation of the vapour [32]. Unselected chronic cough patients had heightened glottic stop responses to ammonia, but responses in sub-groups of patients were not reported and very few subjects in the study had GORD related cough [33]. However, it must be born in mind that such challenges are likely to evoke both pharyngeal and laryngeal reflexes as the ammonia vapour cannot be directed at a single receptive field.

Numerous studies have also documented that patients with chronic cough also have exaggerated cough responses to a variety of inhaled irritants (e.g. capsaicin and citric acid) when compared with healthy controls [34]. Whilst this hypersensitivity is generally interpreted as part of the mechanism responsible for excessive coughing, it is likely to also provide enhanced airway protection.

#### Airway pepsin and bile acids

As an alternative to exploring airway protective reflexes, several investigators have sought evidence of refluxate entering the airways by the detection of components of gastric fluid. Most commonly pepsin and bile acids have been measured in samples from the proximal (sputum) and more distal airways (bronchoalveolar lavage (BAL)). Whilst there is on-going debate about whether the assays are reliable and which enzymes in refluxate are most damaging to the airways, all studies that have included patients with chronic cough, have failed to demonstrate increased airway pepsin or bile acids compared with healthy controls in sputum or BAL [19,35-37]. Of note, pepsin concentrations in induced sputum samples from chronic cough patients were found to negatively correlate with objective cough frequency, unlike lavage fluid pepsin, consistent with cough protecting the airways from pepsin entry.

#### Summary of direct mechanisms

In summary, current evidence supporting direct provocation of coughing by refluxate stimulating the pharynx/larynx or airways is scant in patients with chronic cough. Studies measuring the number of reflux events extending in to the proximal oesophagus or pharynx suggest that, as in healthy controls, very few such events occur. Pharyngeal and laryngeal reflex responses have rarely been studied in chronic cough patients, but it would seem unlikely that impairment of the laryngeal adductor reflex confers a significant aspiration risk, especially in the context of normal swallowing and heightened cough and glottic stop responses to chemical stimuli. Indeed several studies have indicated that very little pepsin or bile acid is present in the airways of patients with chronic cough.

### Indirect mechanisms

The shared vagal innervation of the oesophagus and airway and convergence of vagal afferents in the brainstem provides a means via which reflux events confined to the oesophagus may provoke airway responses in the form of bronchoconstriction and cough.

#### Temporal associations between reflux and cough

Most recent studies using pH/MII monitoring have suggested that patients presenting with chronic cough have physiological numbers of reflux events and oesophageal acid exposure with the vast majority of events being confined to the distal oesophagus (80%) [19-21]. Nonetheless, a growing number of studies suggest that in a significant number of patients coughing episodes seem to be temporally linked to reflux events [20,21,38-40]. These studies have shown that between 20 and 48% of patients has a positive symptom association probability (SAP), for reflux preceding cough i.e. reflux-cough associations. The SAP is calculated assuming reflux and cough events are linked if they fall within 2 minutes of one another, and the Fisher exact test is applied to assess whether the number of linked events exceeds that expected by chance alone. A SAP of 95% or greater is assumed to indicate a positive association [41]. The appropriateness of the 2 minute window however needs further exploration.

As might be expected, studies using ambulatory acoustic systems to record cough sounds [38,39] have reported greater prevalence for both reflux-cough and cough-reflux associations than other techniques, such as manometry [20,21,40]. This is probably because it easier to identify single coughs using acoustic systems than manometry, where a single rapid pressure changes could represent either cough or artefact, such as clearing of the throat, and thus be rejected on a cautionary level by the observer. Studies relying upon the patient to press an event marker to document coughing episodes are the most unreliable, missing 71% of audible cough events [42,43] and 61% of manometric events [21].

We have recently shown using synchronized pH/impedance and objective acoustic cough recording that over 70% of patients exhibit temporal relationships between cough and distal oesophageal reflux, with 48% having a positive SAP for reflux-cough associations (SAP_R-C_), 56% having a positive SAP for cough-reflux associations (SAP_C-R_), and 32% both, raising the possibility of a self-perpetuating cycle maintaining chronic cough [38]. Despite slightly more patients having a positive SAP_C-R_ than SAP_R-C_, those with a positive SAP_C-R_ had fewer temporally linked events, suggesting that reflux triggering cough occurs more often than cough triggering reflux. Notably as in other studies, we also showed that the pH of the reflux event linked to cough was not important, and that non-acid events were as likely to be linked to cough as acid reflux events.

It is often assumed that the straining that occurs during coughing can be responsible for provoking reflux events, explaining cough-reflux associations. Reduction of the time window from 2 minutes to 10 seconds for the SAP calculations significantly reduced the percentage of patients exhibiting a positive SAP_C-R_ to 24%, accounting for only 6% of reflux events_,_ suggesting as in previous studies [44-46] that straining is not a major factor in reflux.

#### Oesophageal-bronchial reflex

The observation that coughs temporally associate with preceding distal oesophageal reflux, supports a neuronal mechanism linking reflux to cough. Neuronal oesophageal-airway crosstalk most likely occurs where vagal afferents converge and synapse within the brainstem in the nucleus tractus solitarius (nTS). This has previously often been described as the “oesophago-bronchial reflex”. Further evidence supporting the presence of such a mechanism comes from studies infusing acid into the distal oesophagus reporting increased cough frequency [47] and cough reflex sensitivity [48] in patients with chronic cough and associated GORD. Oesophageal acid infusion has not been found to change cough reflex sensitivity in healthy volunteers [48], suggesting that this pathway is usually dormant under normal physiological conditions. This might imply that patients exhibiting a positive SAP_R-C_ have a central sensitisation or neuroplastic change in these pathways permitting oesophageal stimuli to modulate airway responses, which is in keeping with the finding that SAP_R-C_ positive patients have heightened cough responses to inhaled citric acid compared with negative SAP_R-C_ subjects. Indeed a difference in cough reflex sensitivity was the only factor that discriminated between these groups, as the number and acidity of reflux events and incidence of oesophagitis at gastroscopy, were no different between groups. Only in one study in patients with suspected GORD presenting with otolaryngology symptoms has cough been suggested to be associated with oesophagitis [49]_,_ but identification of such disease appeared to be a poor predictor of treatment response [50]. Other concomitant conditions potentially contributing to cough, such as nasal disease, asthma, eosinophilic bronchitis and bronchiectasis also had similar prevalence in SAP_R-C_ positive and negative subjects, suggesting reflux may contribute to coughing independently of other conditions.

Exactly how vagal pathways might become modified to permit oesophageal-airway crosstalk is not known, but there is evidence that similar processes are also present in other conditions. For example, in asthma patients several studies found that oesophageal acid infusion can induce bronchoconstriction [51-54] and also cough reflex hypersensitivity [55]. However a meta-analysis of studies of PPI in asthma only found a small improvement in peak flow, unlikely to be clinically important [56]. Experimental oesophageal acid infusion studies such as these have contributed to the expectation that acid suppression therapy should be successful in patients with cough/asthma and GOR, but it should be acknowledged that the responses to oesophageal acid may be a reflection of a more general visceral hypersensitivity of the oesophagus, which may occur in response to a variety of stimuli and therefore does not necessarily implicate oesophageal acid in the pathophysiology.

Sensitization within the nTS of the brain stem may also be responsible for the reverse association i.e. cough induced reflux. Vagal afferents from the proximal stomach are responsible for transient lower oesophageal sphincter relaxation (TLOSR), the main mechanism thought to be responsible for reflux events in both health and disease [57,58]. These afferents could conceivably be activated by cough induced diaphragmatic motion, or by changes in vagal tone associated with cough reflex activations to initiate TLOSRs and thus reflux events, even several minutes after cough [38].

#### Summary of indirect mechanisms

In summary, several studies from different laboratories using MII/pH monitoring agree that intra-oesophageal reflux in patients with chronic cough has similar characteristics to that in healthy populations. However these same studies have found statistically significant temporal associations, suggesting cough follows reflux events more frequently than would be expected by chance alone in up to 50% of patients and independent of pH of the refluxate. The most likely explanation for these associations is crosstalk between airway and oesophageal afferents at their site of convergence in the brainstem, suggesting in such patients, central nervous system mechanisms are responsible for chronic cough.

### Implications for treatment of gastro-oesophageal reflux in chronic cough

#### Proton pump inhibitors

Given the available evidence, it is not surprising that acid suppressants have demonstrated little overall therapeutic effect in most patients with chronic cough. Reducing the acidity of reflux events in patients with positive reflux-cough associations may be insufficient to prevent these events provoking cough. Nonetheless, it remains possible that a sub-group of patients might respond to an appropriate dosing regimen and duration of treatment with such medications; the trials of PPIs performed to date have all been of insufficient size to identify such a sub-group. A recent systematic review re-evaluated the response of chronic cough to antacid therapy in trials that included objective evidence of oesophageal acid exposure on 24 hr pH monitoring [59]. Therapeutic gain was greater in patients with evidence of pathological acid reflux (range 12.5–35.8%) than in those without (range 0.0–8.6%), providing some evidence for the notion that failure to include those patients most likely to respond to acid suppression may have contributed to negative findings in some trials. Indeed as most studies have recruited subjects from secondary and tertiary care, the sub-group of patients responsive to PPIs could be poorly represented as a consequence of successful treatment in primary care.

It remains to be seen whether patients with excessive oesophageal acid exposure and positive reflux-cough associations have the best chance of responding to PPI treatment. Future studies need also to be cognisant of the problems with carryover effects observed in crossover studies of PPIs [6] and that objective cough counts are much more powerful than subjective scores for detecting treatment effects and identifying responders from non-responders [60].

#### Reflux inhibitors

Several novel compounds have recently been shown to reduce the number of gastro-oesophageal reflux events in both healthy controls and patients with reflux disease by inhibition of TLOSRs [61] but none to date have been assessed in patients extra-oesophageal reflux symptoms such as chronic cough. The modes of action of reflux inhibitors include gamma-aminobutyric acid B (GABA_B_) receptor agonism and metabotropic glutamate receptor 5 (mGlu5) antagonists. The most extensively investigated reflux inhibitor has been the GABA_B_ receptor agonist Lesogaberan, but unfortunately efficacy has been disappointing as an add-on therapy to PPIs in patients with typical symptoms of GORD [62,63]. Lesogaberan shares its mechanism of action with Baclofen but with its main activity in the peripheral rather than the central nervous system, reducing side effects. Baclofen is thought to improve symptoms in patients with chronic cough [64,65], which could be by inhibiting reflux [66] and/or by reducing the sensitivity of the cough reflex [67]. Recent pre-clinical data suggests despite a peripheral mode of action, Lesogaberan may have similar inhibitory effects to Baclofen on experimentally induced cough [68]. It is currently unknown whether reflux inhibitors may prove to have clinically important therapeutic benefit in patients with chronic cough; nevertheless, they provide a useful tool to further investigate the associations between cough and reflux.

#### Inhibition of the oesophago-bronchial reflex and central sensitisation

Perhaps the most logical approach to treating reflux related cough might be to target the central neuronal sensitisation responsible for reflux-cough associations, but at present we know little about the relevant neuronal targets. In pain hypersensitivity states, the N-Methyl-D-aspartate *(*NMDA) receptor has been implicated in the development of central sensitisation. Whilst dextromethorphan, a NMDA receptor antagonist, is one of the commonest over the counter medicines used to treat cough, its efficacy is poor [69]. Interestingly, the anti-tussive effect of this class of agents seems to vary significantly between agents [70] and we have found intravenous ketamine to be ineffective in chronic cough patients [71]. It is possible though that some of the centrally acting agents with evidence to suggest efficacy in refractory cough, such as morphine and gabapentin [72,73], may exert some of their effect by reducing cough responses to oesophageal reflux, but mechanistic studies have yet to address this possibility.

#### Surgical procedures

Laparoscopic fundoplication is as effective as long term pharmacological treatment for patients with typical symptoms of reflux disease responsive to PPI treatment [74]. Surgical intervention for reflux in chronic cough is more controversial as atypical symptoms, normal acid exposure and persisting symptoms on PPI therapy have all been associated with poorer outcomes [75]. Fundoplication reduces both acid and non-acid reflux [76] and therefore has the potential to reduce coughing provoked by reflux events. Whilst many studies have reported series of patients with a variety of atypical reflux symptoms, fewer have specifically focussed on patients solely with chronic cough. The majority of reports are retrospective reviews of outcomes for clinical procedures in patients undergoing surgery for chronic cough resistant to PPI therapy [77-80]. Most investigators selected subjects with reflux-cough associations based on patient registration of coughs, now known to be unreliable [78,81-84]; some selected those with abnormal oesophgeal acid exposure [79,85] and one used the patients history alone [80]. Complete resolution of the cough was reported in about 60% of subjects, compared with >90% in patients with typical reflux [75]. These studies do not identify clear predictors of a good response to surgery and rates of post operative dysphagia were high in some series, ranging 19-66%. It remains to be determined whether pH/impedance parameters or acoustic reflux-cough associations might shed light on which patients may benefit most from surgery.

## Conclusions

Considerable progress has been made in recent years towards a better understand of the processes which may permit gastro-oesophageal reflux events to influence cough. Similar work is needed in other respiratory conditions if we are to begin to understand whether reflux disease has a significant influence. The current body of evidence largely supports the concept that neuronal crosstalk between the oesophagus and airway drives the relationships between gastro-oesophageal reflux and cough, however present technologies limit our ability to accurately detect refluxate reaching the larynx, pharynx and lower airways. Future strategies for treating patients with cough reflux associations are likely to include treatments to modulate the numbers of reflux events or the underlying neuronal hypersensitivity.

## Competing interests

JAS is a named inventor on a patent describing novel methods for cough detection and collaborates with a medical device company in the development of a cough monitoring system (VitaloJAK). LAH has no competing interests.

## Authors’ contributions

JAS and LAH both contributed to writing the manuscript and all authors read and reviewed the final version.

## Funding

MRC Clinician Scientist Award G0701918.
